# Does rural transformation affect rural income inequality? Insights from cross-district panel data analysis in Bangladesh

**DOI:** 10.1016/j.heliyon.2024.e30562

**Published:** 2024-04-30

**Authors:** Al Amin Al Abbasi, Subrata Saha, Ismat Ara Begum, Maria Fay Rola-Rubzen, Andrew M. McKenzie, Mohammad Jahangir Alam

**Affiliations:** a Department of Agribusiness and Marketing, Bangladesh Agricultural University, Mymensingh, 2202, Bangladesh; b Department of Agricultural Economics, Bangladesh Agricultural University, Mymensingh, 2202, Bangladesh; c Center for Agricultural Economics and Development, UWA School of Agriculture and Environment, University of Western Australia, Australia; d Department of Agricultural Economics and Agribusiness, University of Arkansas, Fayetteville, AR, 72701, USA

**Keywords:** Rural transformation, Income inequality, Gini coefficient, Bangladesh

## Abstract

Rural transformation plays a crucial role in enhancing the income and employment prospects of the rural labor force. We investigate the effects of rural transformation on rural income inequality at the district level in Bangladesh using data from five years of nationally representative Household Income and Expenditure Surveys. The Gini coefficient is used to measure rural income inequality. In contrast, the share of high-value agricultural outputs and the share of rural non-farm employment are used as indicators of rural transformation. We find that rural income inequality is positively associated with the share of high-value agricultural outputs and the share of rural non-farm employment. The non-linear regression result shows an inverted U-shaped relationship between rural transformation and income inequality, which indicates that income inequality initially increases with rural transformation but decreases in the long run. Additionally, we find that rural income inequality is positively correlated with the proportion of household education expenditures, agricultural rental activity, and the share of remittances. This study also reveals that income inequality in rural areas of Bangladesh has a significant negative correlation with the government's social safety net program.

## 1 Introduction

Rural transformation (RT) is characterized by increased agricultural output, commercialization, surpluses that may be sold, and diversification of production patterns and livelihoods [1]. In addition, it involves the expansion of decent off-farm employment and entrepreneurial prospects, improvement of rural coverage and access to services and infrastructure, and the expansion of access to and influences over important governmental processes [2]. Rural transformation results in widespread rural expansion and better-managed, more sustainable rural landscapes [[3], [4], [5]]. Many rural areas in the Asia-Pacific region have recently undergone transformation, including gross domestic product (GDP) growth, shifts from grain-based agriculture to higher-valued agricultural outputs, and employment shifts from agriculture to other sectors [3]. For instance, the percentage of rural non-farm income as a share of total income in China increased from 34 % in 1985 to 63 % in 2000 and 71 % in 2010 [6]. In the 1990s and 2000s, non-farm income accounted for 37 % of total household income in Africa, compared to 47 % in Latin America and 51 % in Asia [7]. With a GDP growth rate of 7 % in 2019 and an increase in per capita GDP from US$ 518 in 1991 to US$ 1715 in 2021, Bangladesh, too, is undergoing a rural transformation process [8]. This change is evidenced by World Bank data indicating that non-farm employment increased from 30 % in 1991 to 62 % in 2019 [9]. There has also been an increase in diversification from basic staples to other agricultural commodities. For example, the production value share of livestock, fisheries, and spices has increased from 6 % in 1991 to 17 % in 2020 [10]. Moreover, the production of cash crops, particularly vegetables and fruits, has increased more rapidly than grain production. Despite having one of the most promising economies in the 21st century, Bangladesh has one of the highest income inequality rates in Asia, with the population's top 5 % income earners capturing 95 % of total income, indicating an unequal distribution of wealth [11]. Because of rising income inequality, a significant portion of the population is not receiving the benefits expected from the rapidly expanding economy.

Extensive research has been done on the relationship between economic determinants, changes in rural structure, and income inequality from different perspectives to comprehend the wide-ranging social effects of agricultural and rural development. Meschi and Vivarelli [12] found that trading with high-income countries increases income disparities in developing nations, affecting imports and exports. Technological gaps and skill-biased technologies caused this. Importantly, these findings mostly apply to middle-income countries, where technological upgrading might have increased economic inequality. Topuz and Dağdemir [13] identified a non-linear U-shaped relationship between trade liberalization and income inequality in Turkey and suggested that financial development, income per capita, and internal trade helped to reduce income inequality. In the United States, Butler et al. [14] found that declining rural population levels raise income inequality, whereas growing population levels slightly lowered it. Geographic location, baseline inequality levels, and baseline population size affected the relationship between income inequality and population change. Suesse and Wolf [15] found that structural restructuring causes credit cooperatives to arise from dropping agricultural staple prices and declining bank lending. Land inequality and low asset sizes hindered regional cooperative growth, while ethnic heterogeneity had mixed consequences. In Sub-Saharan Africa, specifically in Zimbabwe, Tanzania, and Mozambique, Manero [16] investigated income disparities within smallholder irrigation schemes, and results showed that income disparities within these schemes were significantly higher than national figures, highlighting the necessity for region-specific development policies. Zerihun [17] examined the influence of non-farm income diversification on household welfare and income equality in rural Ethiopia and concluded that non-farm income improved the wellbeing of rural households while also raising overall income equality. Sun and Wang [18] found that after controlling for absolute income, household spending was inversely correlated with relative income position and positively correlated with rural income disparity, indicating a complicated interaction in China. Mukhopadhaya [19] detailed China's rising income inequality due to economic growth, urbanization, and labor market changes. Wages, pensions, and transfers are the main causes of this inequality, and government programs are ineffective. Xu et al. [20] found that income from household operations promoted rural income inequality in China's southwestern mountainous region. Transfer income regularly reduced income inequality, while wage income initially reduced inequality but reversed its effect after an earthquake. Do et al. [21] found that positive livestock income in rural Vietnam increased annual household income and reduced rural income inequality, but negative livestock income had the opposite effect. Doan et al. [22] studied wage inequality in Vietnam and showed that it increased initially but reduced significantly afterwards. The increased supply of higher-educated labor and economic restructuring shaped wage inequality. Rauf et al. [23] claimed that throughout Indonesia, regions and households experienced a moderate degree of income inequality that exhibited a propensity for variation. The findings also indicate that the mining and industrial sectors had been successful in countering the agricultural sector's dominance. Rahut et al. [24] studied rural nonfarm employment, income, and inequality in Bhutan and found that rural nonfarm activities contributed significantly to rural household income. Education and labor supply helped access more remunerative nonfarm jobs, whereas petty self-employment reduced inequality. According to Iqbal et al. [25], non-farm income helped to reduce poverty, while an increase in non-farm income marginally raised income disparity among households in the Punjab province of Pakistan. In North-east India, Priscilla et al. [26] observed that increasing earnings transfer crop farming to more profitable non-farm activities. Although agriculture is the main source of revenue, it reduced economic inequality, and intra-state, intra-district, and landholding size discrepancies drive regional income disparity. Das and Srivastava [27] focused on income inequality in Indian agricultural households and found that Income disparity and land inequality are highest in states with developed agriculture. Studies reveal that agricultural production is the factor that most significantly influences the disparity in income between farming households, after land size. Wage and livestock income also contribute to reduce overall inequality among agricultural households. In Punjab, India, Saini and Kaur [28] found a severely skewed income distribution among farm households with a high Gini coefficient. Livestock and informal wages reduced income disparity for less-educated and resource-poor farmers, who are more inclined to follow non-agricultural and disorganized income streams. Saini et al. [29] studied income inequality among small farm proprietors in Indian Punjab and found that it rose due to a lack of resources and services.Historically, Bangladesh's economy has been dominated by agriculture and related sectors, and most rural people were economically dependent on agricultural activities. However, over the past two decades, income and employment circumstances have changed, partly due to the rise of the rural non-agricultural sector [30]. There is evidence that adverse climatic conditions that affect agricultural productivity, such as floods and droughts, have a substantial beneficial impact on the growth and transformation of non-farm employment in Bangladesh [31]. Sen [32] demonstrated a rural employment shift in Bangladesh, stressing the rise of non-agricultural jobs in the East due to improved connectivity and microfinance. Sen et al. [33] emphasized the shifting employment patterns of rural Bangladeshi households toward non-agricultural pursuits and the emergence of mixed households, where urban proximity is a major factor. In particular, Deb [34] emphasized the remarkable agricultural expansion in Bangladesh, with a specific focus on non-crop cultivation and high-value crops. Agriculture continues to be the leading source of employment in Bangladesh, indicating a substantial transformation in the agricultural sector that will have extensive implications for both the economy and employment. The growth in high-value agricultural product output, in turn, helped the sector grow at a faster rate [35]. In their study of coastal Bangladesh, Jamal et al. [36] addressed the topic of subsistence adaptation to climate change. Despite being more profitable, vegetable-based systems entail labor-intensive processes, market entry restrictions, environmental hazards, and increased expenditures. These challenges imply the need for climate resilience strategies and initiatives tailored to smallholder farmers.

Khan [37] found that farming, wages, and rental income, which reduce rural income disparity, have either remained the same or dropped as a share of overall income. Income inequality is rising due to salaries, non-farm enterprises, remittances, and property revenue. Growing remittances and salaries exacerbate economic inequality. Osmani and Sen [38] found that while consumption inequality remained stable, income inequality increased in rural Bangladesh. The shift was ascribed to the growth of microcredit initiatives and foreign remittances, which provided insight into the intricacies of rural inequality in the area. Another study by Zaman and Akita [39] emphasized the importance of education for urban inequality, formal incomes for reducing inequality, expanding transfer programs for the poorest, and stimulating agricultural productivity and non-agricultural activities in Bangladesh to reduce income disparities and poverty. According to Matin [40], an increasing Gini concentration ratio at various levels in Bangladesh indicates a persistent income transfer to higher income groups and a growing income disparity. The study emphasized that to reduce economic inequality, technical and vocational education and training (TVET) should be included in national policy, and agriculture should remain the primary source of employment.

Bayes and Hossain [41] identified several reasons for rural income disparities in Bangladesh. These include the high capital requirements of non-rice and non-crop agriculture, which the poor cannot readily acquire; large farmers producing high-value crops commercially; and increased non-agricultural income, which enhances larger inequality. Hossain et al. [42] found that non-farm income reduced rural poverty in Bangladesh but increased expenditure inequality. They revealed that education, remittances, power access, and high-return sector involvement affected household non-farm income. Alamgir et al. [43] revealed significant per capita income disparities among farm households in flood-prone areas of Bangladesh due to agricultural income variations. Higher agricultural income reduced income inequality in these areas, highlighting the repercussions of climate change on farming communities.

The comprehensive literature analysis offers valuable insights into the intricacies of income inequality in rural areas, specifically examining multiple countries and locations. Further investigation is required to take into account the distinct socio-economic and environmental circumstances of each region. The majority of the research that has been evaluated is either cross-sectional or has been conducted within defined time frames. The literature study mostly offers insights on a national scale (see Refs. [30,[33], [34], [35],^39^,^40^]). However, there is a research gap in doing micro-level studies within rural parts of Bangladesh, and there is a need for more comprehensive analysis specific to districts. Several studies emphasized the presence of income inequality specific to certain areas (see Refs. [37,38,41,42]). However, it is important to note that income inequality in rural areas and the effects of rural transformation can differ greatly among districts due to various economic activity, demographic factors, and policy interventions. Improving understanding of these differences would be highly beneficial in facilitating comprehension of how income inequality materializes at the local level and how regional circumstances impact it. Currently, there is a lack of studies that specifically examine longitudinal analysis in rural areas, resulting in a research gap that overlooks the changes in income disparity and its underlying factors over time in Bangladesh. To gain a thorough comprehension of income inequality in rural areas, it is necessary to conduct a more extensive analysis over a longer period, taking into account the changing economic landscape and shifting employment patterns in the country. Climate change impacts in Bangladesh have the potential to greatly influence economic disparity, as indicated by several research (see Refs. [31,36,43]). However, there are areas of inquiry that have not been explored in the existing literature about the effect of rural transformation on income inequality in a country that heavily depends on agriculture.

This study fills important gaps in the literature on income inequality in Bangladesh's rural areas, which makes it noteworthy. Its novelty includes examining the effects of rural transformation on income inequality—a topic frequently ignored in prior research—and doing household-level analysis within particular districts. The study also uses a longitudinal approach to capture changes over time. The study also focuses on the consequences of rural transformation in a nation highly dependent on agriculture, offering useful insights for developing policy. The research attempts to deliver robust and solid insights by focusing on developing causal links, giving policymakers a more sophisticated nuance of the issues surrounding income disparity. The results of this study will contribute to the development of focused and efficient policies that address the unique issues raised by Bangladesh's rural transformation and income inequality. This will help to reshape the strategies described in the Annual Development Programs (ADP) and Five-Year Plans of the country. Furthermore, at a global level, the research findings may provide valuable insights to scholars and policymakers dealing with similar issues in other agrarian economies, particularly in South Asian countries experiencing stages of rural transformation like Bangladesh. A comprehensive understanding of how rural change affects income disparity provides a universally applicable perspective. To promote a more inclusive and equitable global approach to rural development, researchers and policymakers worldwide can utilize the study's methodology and findings and adapt programs addressing income inequality in their rural contexts.

In this paper, we investigate trends in income inequality, rural transformation, and the spatial-temporal pattern of income Gini coefficients in Bangladesh and estimate the impact of rural transformation on income inequality in the country. The remainder of this paper is structured as follows. In Section 2, we present the indicators of rural transformation and other control variables. Section 3 details the research methods, which include the data collection and analysis processes, statistical methods, and econometric models used in this investigation. Section 4 presents a descriptive analysis of rural transformation and income disparity indicators and results, as well as a discussion of estimated models, including pooled and fixed effects. The last section provides the conclusion and policy implications.

## 2 Indicators of rural transformation and control variables

It is crucial to emphasize that the concept of rural transformation covers various issues, including economic and social developments in rural areas. Despite the fact that debates on rural transformation frequently center on the economic impact of the agricultural revolution, it is important to understand that it also entails more extensive transformations [5,44]. According to IFAD [3], a rural transformation process involves more opportunities for off-farm employment and more diverse and marketable agricultural products. In this research, we define rural transformation as a process whereby the rural labor force moves from farm work to other sectors as the productivity of agricultural labor increases and the farming production structure shifts from grains and other staple food crops to more diverse and marketable high-value agriculture. In light of this, shifting production from inexpensive to high-value crops and expanding off-farm employment in rural regions are crucial for rural development. Horticulture, cattle, and fisheries are examples of high-value agriculture in the agricultural industry [45]. Typical low-value crops include grains, cotton, and sugar. We use the ratio of high-value agricultural goods (fruits, vegetables, livestock, and fisheries) to the total output value of agriculture (which includes all crops, livestock, and fisheries), which is known as RT1, as an indicator of RT.

Another indicator of rural transformation is the shift from agricultural to non-agricultural employment. In addition to the shift in agriculture, rural employment in Bangladesh has undergone a remarkable transformation over the past few decades, shifting from agriculture to non-agriculture. In this study, we consider the change in rural employment as a share of rural non-farm employment as another indicator of RT (RT2).

It is important to consider other control variables that have been studied in the past when examining how rural development influences income inequality. A key control variable is the fraction of household education spending, calculated by dividing it by their total expenditure. According to a Portuguese household survey, education reduces inequality at all income levels but not at expenditure levels [46]. In India, growing employee education returns did not increase household inequality. Due to slower illiteracy, education inequality has led to income inequality [47]. Other research shows that education and income inequality are positively correlated in high- and low-income countries but negatively correlated in middle-income countries [48].

Another control variable is the share of household healthcare expenditure, calculated by dividing it by household expenditure. In the US, healthcare costs have increased income disparity and impoverished millions. Health costs lowered the average income in the poorest deciles and pushed 7.013 million people into poverty [49]. Income inequality in household health expenditures in India caused caste-based health care disparities. Hospitalization costs were most economically damaging, especially for backward caste families [50].

Farmland rental activity, determined by the ratio of rented land to household farmland, is another control variable. WenJing et al. [51] found that Chinese households were more likely to rent farms and increase agricultural production when their farm income is higher. Farmland was transferred to more productive farmers in Vietnam. The fact that market activity does not benefit impoverished households highlights the need to help them [52]. Farmland rental activities affect agricultural revenue and inequality.

Remittances significantly impact rural income. The share of remittance is another control variable and is determined by its portion of rural income. Remittances cause economic disparity compared to non-remittance recipients, but they also diminish inequality within the recipient group [53]. Foreign and domestic remittances have reduced poverty, depth, and severity in Sri Lanka, although income inequality has decreased slightly [54]. Across Kenya, remittances increase household spending, especially for poorer families [55].

Government social safety net coverage of rural households is another control variable. According to Turnovsky and Erauskin [56], government investment has both positive and negative effects on income inequality. In China, government transfers and relief payments reduce inequality and poverty, but not the poorest [57]. In southern Africa, elderly social assistance has reduced poverty but not income inequality, reducing the direct effect of social welfare benefits on inequality [58].

## 3 Methodology

### 3.1 Data sources

In this research, we used data from five rounds of the Household Income and Expenditure Survey (HIES): 1995, 2000, 2005, 2010, and 2016. The survey was carried out by the Bangladesh Bureau of Statistics (BBS); hence, the data are nationally representative. The dataset was purchased by the research team from BBS. Bangladesh is divided into eight divisions, and the divisions are divided into 64 districts. At the district level, we calculated the Gini index, RT1, RT2, and other control variables using household data. In this study, we compiled every round of HIES data for 64 districts for 320 observations. Table 1 shows the data used and the sources of the data.

**Table 1:** Summary of variables.

Variable	Description	Units
Rural income inequality	Measured by Gini coefficient	Proportion
Share of high-value agriculture	Total high-value agricultural product value of the district (including fruits, vegetables, livestock, and fisheries) divided by the total agricultural product value of the district.	Proportion
Share of rural non-farm employment	Ratio of the total number of rural non-agricultural employment in the district to the total number of rural employments.	Proportion
Share of household education expenditure	Sum of households' education expenditure divided by the sum of total expenditure.	Proportion
Share of household healthcare expenditure	Sum of households' healthcare expenditure divided by the sum of total expenditure	Proportion
Farmland rental activity	Ratio of the amount of rented-out cultivated land to the total amount of farmland owned by the household	Proportion
Share of remittance	Proportion of money sent by immigrants to total rural income	Proportion
Share of rural households under the Social Safety Net program	Proportion of rural households covered by the government's social safety net program of total rural households	Proportion

### 3.2 Estimation of lorenz curve and gini coefficient

The two most popular techniques for examining income inequality are the Lorenz curve and the Gini coefficient. The graphic representation of the income distribution is the Lorenz curve [59]. The region bounded by the Lorenz curve and the egalitarian line, representing complete equality as a line at a 45-degree angle, is two times the Gini coefficient. However, it is crucial to acknowledge the limitations of the Gini coefficient when considering it as the optimal measure of income inequality. An important limitation is the inability of the Gini coefficient to differentiate between various forms of inequality. Despite variations in Lorenz curves, which may intersect and indicate diverse patterns of income distribution, the resulting Gini coefficient values can be surprisingly similar [60]. This attribute introduces complexity into the process of comparing Gini coefficient values and could potentially present challenges when attempting to test hypotheses pertaining to income inequality. Furthermore, researchers should be aware that the Gini coefficient is sensitive to middle-income differences. While suitable for many studies, sometimes it is better to focus on inequalities at the high or low ends [61,62]. This shows that the Gini coefficient is not completely neutral and is just one way among many to measure income inequality. Nonetheless, the Gini coefficient remains one of the most popular measures of income inequality. The steps involved in determining the Gini coefficient are as follows: grouping districts according to the income size of the households in order of lowest to highest; determining the gross income of each district; determining the cumulative proportion of rural households; and determining the cumulative proportion of household's income for each district. Finally, the Lorenz curve is created. The Lorenz curve is used to infer the Gini coefficient [63].

(1) G=1−∑[a×(2b−c)]

where *G* is the Gini coefficient, *a* represents the share of rural households, *c* is the gross income per household, and *b* is the cumulative proportion of gross income per household. We used this formula to determine the Gini coefficient for 64 districts. The Gini coefficient has a range of 0–1. If the distribution of household income in each district becomes more unequal, the Gini coefficient rises, and the opposite is also true.

### 3.3 Spatial data analysis

The Getis-Ord Gi* tool is used to study the patterns of Gini coefficients to identify the precise location of rural income inequality in Bangladesh. According to Songchitruksa and Zeng [64], the Getis-Ord Gi* index is used to calculate spatial aggregation, which indicates if features with high (low) or low (high) values are located around a feature with a high (low) value. The Getis-Ord Gi* statistic is useful for identifying spatial autocorrelation but faces limitations such as the inability to identify negative spatial autocorrelation and weak significance tests, which may result in false positives. Limiting the search radius to tackle important issues has a practical threshold, resulting in a trade-off between precision and accuracy [[65], [66], [67]]. For a more thorough interpretation, it is recommended to incorporate complementary metrics. Caution should be used when analyzing big *G* values, and zones with the highest or lowest values should be the emphasis for enhanced reliability. We explore spatial dimensions of income inequalities using hot spot analysis of the Gini coefficient across districts and inferential statistics, meaning that the analysis results are interpreted with respect to the null hypothesis. The Getis-Ord Gi* and the hypothesis are indicated as

(2) $$G_{i}^{*}=\frac{\sum_{j=1}^{n}w_{i,j}x_{j}-\bar{X}\sum_{j=1}^{n}w_{i,j}}{S\sqrt{\frac{\left[n\sum_{j=1}^{n}w_{i,j}^{2}{\left(\sum_{j=1}^{n}w_{i,j}\right)}^{2}\right]}{n-1}}}$$

(3) $$\bar{X}=\frac{\sum_{j=1}^{n}x_{j}}{n}$$

(4) $$S^{2}=\frac{\sum_{j=1}^{n}x_{j}^{2}}{n}-\;{\left(\bar{X}\right)}^{2}$$

H_0_: Distribution of the Gini index at the district level is random.

H_a_: Distribution of the Gini index at the district level is clustered.

Where: $G_{i}^{*}$ is a statistic that describes the spatial dependency of location *i* over all *n* districts; $x_{j}$ is the value of the Gini coefficient at incident location *j* over all *n*, and $w_{ij}$ is the weight value between *i* and *j* that represents their spatial interrelationship. $\bar{X}$ is the mean, and $S^{2}$ is the variance of the Gini coefficient. This analysis provides insights into income inequality in rural areas within certain districts. The study intends to determine any association between the intensity of income inequality and the variables RT1 and RT2 in those districts.

### 3.4 Econometric model

We used pooled regression and fixed-effect regression in this study. Pooled regression does not take into consideration unobserved unit-specific characteristics that may have an impact on the outcome variable because it assumes that all units have the same intercept and slope. The following is a pooled regression model:

(5) $$G_{it}=\alpha_{0}+\alpha_{1}{RT1}_{it}+{\alpha_{2}RT2}_{it}+{\alpha_{3}SEE}_{it}+{\alpha_{4}SHE}_{it}+{\alpha_{5}RENT}_{it}+{\alpha_{6}SR}_{it}+{\alpha_{7}GSP}_{it}+u_{it}$$

Fixed effects regressions examine how rural change affects rural income inequality. We use panel data with several district observations throughout time. A fixed effects regression controls for time-invariant unobserved district differences in panel data and subtracts the temporal mean from each model variable. The model is written as follows:

(6) $$G_{it}=\beta_{0}+\beta_{1}{RT1}_{it}+{\beta_{2}RT2}_{it}+{\beta_{3}SEE}_{it}+{\beta_{4}SHE}_{it}+{\beta_{5}RENT}_{it}+{\beta_{6}SR}_{it}+{\beta_{7}GSP}_{it}+\upsilon_{i}+u_{it}$$

(7) $$G_{it}=\gamma_{0}+\gamma_{1}{RT1}_{it}+\gamma_{2}{RT1}_{it}^{2}+{\gamma_{3}RT2}_{it}+\gamma_{4}{RT2}_{it}^{2}+{\gamma_{5}SEE}_{it}+{\gamma_{6}SHE}_{it}+{\gamma_{7}RENT}_{it}+{\gamma_{8}SR}_{it}+{\gamma_{9}GSP}_{it}+\upsilon_{i}+u_{it}$$

where *i* denotes the district, *t* denotes the year, and *G* is the Gini coefficient. RT1 is the share of high-value agriculture, and RT2 is the share of rural non-farm employment. SEE stands for share of household education expenditure, SHE stands for share of household healthcare expenditure, RENT stands for farmland rental activity by the household, SR stands for share of remittance, and GSP stands for the proportion of households under social safety net program provided by the government. In Equations (5)–(7), u_it_ is the error term, and α, β, and γ are the coefficients to be estimated; υ_i_ is the district-specific fixed effect for district *i* is represented in Equations 6 and 7, respectively. Equation (7) indicates a non-linear relationship between rural transformation and income inequality.

This study used moments-quantile regression (MM-QR) with fixed effects. The MM-QR method provides a complete understanding of how independent variables affect the dependent variable's conditional distribution, unlike conventional methods. Machado and Santos [68] defined the following assumptions for the MM-QR model with panel fixed-effects:

(8) $$Q_{G_{it}}\left(\tau |X_{it}\right)=\theta_{i}\left(\tau \right)+{\overset{´}{X}}_{it}\beta +{\overset{´}{W}}_{it}\gamma q\left(\tau \right)\;with\;\theta_{i}\left(\tau \right)\equiv \left(\theta_{i}+\alpha_{i}q\left(\tau \right)\right)$$

where G and X are the dependent (income inequality) and independent (RT1, RT2, SEE, SHE, RENT, SR, and GSP) variables, respectively, and W is a vector of known differentiable transformations of the components of X. $\theta_{i}\left(\tau \right)$ is the quantile (τ) fixed effect for panel unit *i* at time *t*, and β and γ are parameters.

## 4 Results and discussion

### 4.1 Descriptive statistics

Table 2 shows information on eight different variables throughout time. These variables include the Gini index, RT1, RT2, the share of education and healthcare expenditure, the share of rented out farmland, the share of remittances, and the share of households protected by the government's social safety net program. Income inequality increases with Gini index values. From 0.352 in 1995 to 0.457 in 2016, Gini index mean values have grown. Overall, income inequality has increased across the country and among households. This may indicate that the richest earners' incomes expanded faster than the overall population's. At RT1, the mean value rose from 0.073 in 1995 to 0.164 in 2016. This could reflect improved farming technology and a focus on high-value commodities. The mean values of RT2 have increased over time, from 0.305 in 1995 to 0.560 in 2016. This may indicate the emergence of economic diversification and a departure from traditional agricultural practices. The mean values of SEE have increased from 0.029 in 1995 to 0.103 in 2016. This indicates that households are investing more in education and human capital development over time, indicating a rise in the priority they place on education. SEE mean values increased from 0.029 in 1995 to 0.103 in 2016. This suggests that households are prioritising education and human capital development over time. The mean values of SHE increased over time, from 0.024 in 1995 to 0.079 in 2016, according to the data. The upward trajectory of healthcare expenditures indicates a growing concern among households regarding their healthcare. The results demonstrate that the mean values of RENT have increased over time, from 0.062 in 1995 to 0.162 in 2016. This could indicate increased land market activity and a transition in land use patterns. Over time, the mean values of SR have exhibited an upward trend, rising from 0.022 in 1995 to 0.062 in 2016. This could indicate an increase in migration and a greater dependence on international remittances. Since 1995, the average GSP value has risen from 0.022 to 0.122. This may indicate increased government support for low-income households and initiatives to reduce poverty. The mean values of all variables have increased over time, showing economic and social development. However, the standard deviation of factors such as education and healthcare expenditure has increased, indicating higher population variance. Income inequality is rising according to the Gini index, which is concerning.

**Table 2:** Descriptive statistics.

Variables	Total (N = 320)		1995 (N = 64)		2000 (N = 64)		2005 (N = 64)		2010 (N = 64)		2016 (N = 64)	
	Mean	Std. dev.	Mean	Std. dev.	Mean	Std. dev.	Mean	Std. dev.	Mean	Std. dev.	Mean	Std. dev.
Gini index	0.409	0.039	0.352	0.017	0.385	0.015	0.415	0.013	0.434	0.009	0.457	0.010
RT1	0.115	0.039	0.073	0.019	0.092	0.020	0.111	0.021	0.135	0.024	0.164	0.026
RT2	0.429	0.101	0.305	0.037	0.359	0.038	0.423	0.040	0.498	0.050	0.560	0.043
Share of education expenditure (SEE)	0.066	0.046	0.029	0.006	0.042	0.009	0.072	0.082	0.080	0.013	0.103	0.018
Share of medical expenditure (SHE)	0.047	0.040	0.024	0.004	0.034	0.005	0.044	0.006	0.055	0.006	0.079	0.078
Share of cultivated land rent out (RENT)	0.109	0.041	0.062	0.017	0.084	0.018	0.108	0.020	0.131	0.023	0.162	0.026
Share of remittance (SR)	0.053	0.037	0.022	0.011	0.036	0.012	0.062	0.066	0.080	0.014	0.062	0.009
Share of household under social safety net (GSP)	0.065	0.036	0.022	0.003	0.040	0.004	0.059	0.007	0.081	0.009	0.122	0.017

**Fig. 1:**
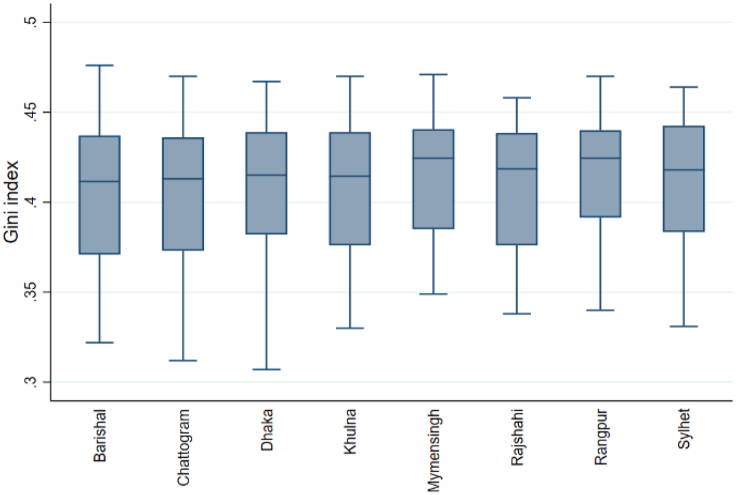
Box plots of the Gini index.

**Fig. 2:**
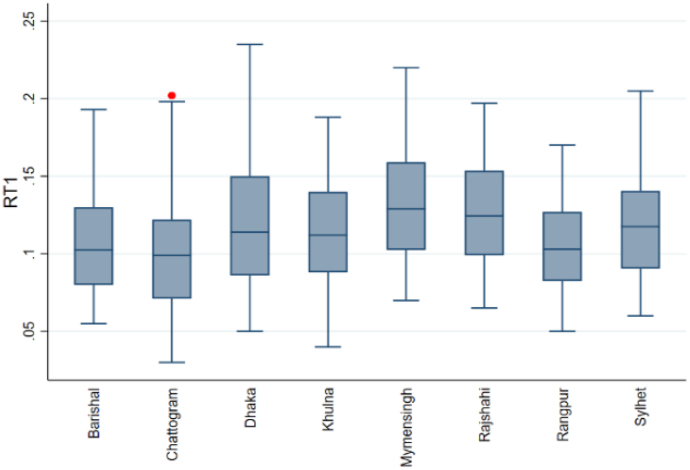
Box plots of RT1.

**Fig. 3:**
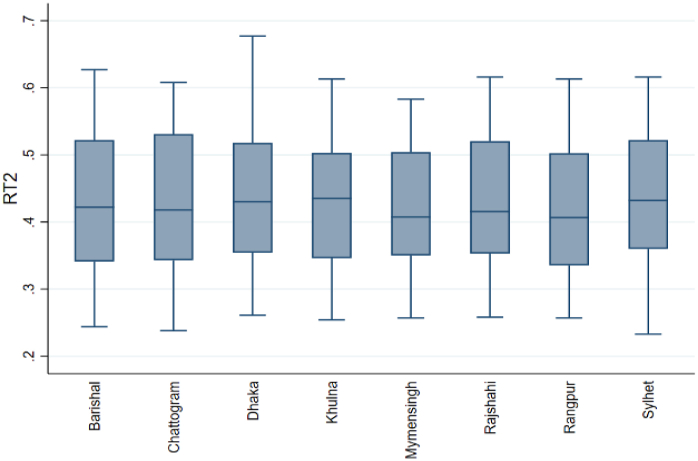
Box plots of RT2.

The box-and-whisker plot shows the distribution of the Gini index, RT1, and RT2 for eight divisions. Box plots show data using the 25th, 50th, and 75th percentiles (Q1, Q2, and Q3). The whiskers are determined by the interquartile range (IQR)= (Q3–Q1). Any values outside 1.5 times the box's IQR are outliers and displayed as points. We examined the box-and-whisker plot to compare the central tendency, variability, and range of the data between the different groups. Fig. 1 shows that Mymensingh and Rangpur have a higher median Gini index than the other six divisions. The box for Mymensingh and Rangpur division is slightly smaller than others, indicating that its middle 50 % Gini index value is more concentrated. Dhaka has the lowest Gini index, while Barishal has the highest, as shown by the longer whiskers. No outliers exist in any division. In Fig. 2, the median of RT1 in Mymensingh is slightly higher than in the other division. The wider boxes and longer whiskers for Dhaka show that the middle 50 % of RT1 value is more spread out than in other divisions. Outliers in the Chattogram division indicate districts with significantly higher RT1. In Fig. 3, the median RT2 is highest in Khulna, followed by other divisions. We have also found that the RT2 range is widest in Chattogram and narrowest in Rangpur, and there are no outliers.

### 4.2 Trends of RT and rural income gini index

This section discusses income inequality and rural transformation trends. The income Gini index (see Equation (1)), RT1, and RT2 trends at the district level are detailed in Appendix A. From 1991 to 2016, all three variables increased in all districts. RT1 has increased due to several reasons, and one of the important reasons is that diversifying output into higher-value crops, livestock, and fishery items has allowed for income growth due to the growing domestic demand for high-value food [69]. According to Dizon et al. [70], urbanization and increasing salaries in Bangladesh are causing a shift in food patterns from grains to nutrient-dense, high-value agricultural products. Additionally, a number of NGOs and international donor organizations are actively promoting agribusiness opportunities, high-value cash crops, and agricultural commercialization in Bangladesh [71]. RT1 increases slower than RT2 and the Gini coefficient. Insufficient infrastructure, credit, financial services, research and development, market linkages, and information can delay high-value agriculture growth [72]. The rise in RT2 in Bangladesh is a result of labor market adjustments to factors like education, financial access, agricultural mechanization, urban proximity, migration, and natural disasters like cyclones, floods, and droughts [73]. Sarkar and Mandal [74] revealed that rural household heads who are educated are more likely to move towards non-farm employment. They also observed a positive correlation between the presence of more earning individuals in a family and the probability of participating in non-farm employment. Small landowners are more likely to participate in non-farm activities, whereas larger farm households can dedicate more time to agricultural production. Improved transportation infrastructure contributes to the rising trend of RT2 by increasing daily wage rates, prompting labor outmigration. This leads to vulnerable day-laborers in farm sectors moving to non-farm jobs with higher marginal returns [75]. The districts of Bandarban, Khagrachari, and Rangamati in the hill-tracks area; Sunamganj, Habiganj, and Kishoreganj in the haor^1^ area; and Barguna, Bhola, Patuakhali, Pirojpur, and Satkhira in the coastal region have slower RT2 trends than the other districts. Landslides, cyclones, and floods in these places might harm infrastructure, slow economic growth, and reduce job expansion. Enterprise reluctance to invest in these areas may limit the expansion of non-agricultural jobs. Low education and poverty in these locations might limit workforce abilities and make it hard for employers to recruit suitable workers. The Gini index exhibits an upward trend but with variations across districts. In developing countries, the increase and deregulation of the financial sector, along with modifications in labor market policy, have led to a growing pattern of income disparity. Inequality is influenced by both unemployment and a rising proportion of low-wage employment [76]. Khan et al. [77] find that in Bangladesh, income inequality is substantially larger than consumption inequality and is rising. Wealth inequality has increased (Gini ratio), and wealth distribution is even more skewed than income. The study also found that urban income and wealth inequality have increased in recent years. There is also a significant difference in wealth between the richest and lowest-class households. Additional evidence indicates that increased revenue from agriculture led to reduced economic inequality in the districts, despite employment income being the primary source of income in most districts. Remittance income exacerbates income inequality in communities with low employment rates and minimal agricultural activity [43].

### 4.3 Lorenz curves by division from 1991 to 2016

**Fig. 4:**
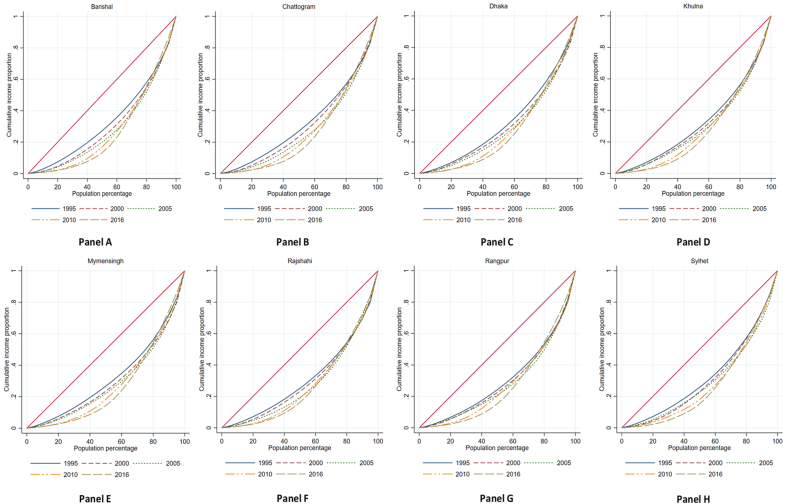
Lorenz curve by division.

Income inequality is explained using the Lorenz curve. Fig. 4 illustrates the cumulative proportion of people on the x-axis (ordered from lowest to highest by income) to their corresponding income on the y-axis. Income inequality in all eight divisions (Panel A–H) increased significantly between 1995 and 2016. Income inequality increased more in the bottom half of 2016. However, the gap between perfect equality and income distribution widens differently between divisions. Also, income inequality is most visible between 10 % and 70 % of the population in all divisions, but in Barishal (Panel A) and Sylhet (Panel H), inequality is more at the bottom level compared to other divisions. The Barishal division is situated in a coastal area that is prone to periodic natural disasters. Poor people are most affected by natural disasters and experience a range of financial setbacks, and have fewer resources, exacerbating the already existing income inequality. According to Ashrafuzzaman [78], most of the infrastructure and livelihood options in the coastal region are exceedingly vulnerable to natural disasters, and increased soil and freshwater salinity, storm surges, and cyclones are the key factors affecting the community's livelihood and income sources. Certain districts in the Sylhet division receive more international remittances than others. In contrast, other districts are geographically disadvantaged due to their location in the Haor area, which could lead to higher income inequality in this division. Khan [37] finds that salaries have nearly tripled since the mid-1990s, yet wages as a percentage of personal income have stayed stable. Foreign remittances and salaries have increased rural income disparity in Bangladesh. The reason may include various factors, including educational and skill development opportunities that are scarce in rural areas. Due to their lack of qualifications for higher-paying jobs, many people are forced to work in low-wage agricultural or manual labor jobs. Another study reveals disparities in facility-based services in Bangladesh, highlighting significant inequalities in utility, education, social welfare, transportation, and health services across different regions [79]. Agriculture pays poorly, and rural industrial and service employment is scarce and competitive; thus, rural laborers work in informal, unregulated jobs with limited benefits. Because of this, many rural workers are pressed to move to cities for better-paying occupations, leaving behind the less mobile or less skilled. Rural areas have unequal land, water, and other resources. Some people struggle to survive, while others have abundant resources.

### 4.4 Correlation between the gini coefficient and rural transformation

The Gini index and RT1 and RT2 are positively correlated (see Appendix B). The slope of the correlation line varies among divisions, suggesting that RT1 and RT2 affect income inequality differently. In rural Bangladesh, where agriculture provides income and sustenance for many households, the Gini index and RT1 connection are essential. Small-scale farmers dominate rural agriculture, using their land for subsistence and income. High-value commodities, including fruits, vegetables, and cattle, can boost profits and economic growth. However, this type of high value agriculture requires more irrigation, seeds, and fertilizers, which may be difficult for small farmers. High-income households in rural Bangladesh have more resources, and more access to credit, markets, and technology, which contributes to income disparity. Therefore, high-value agriculture tends to concentrate in locations with more wealth disparity, where larger farmers and commercial firms have more resources and investment opportunities.

RT2 has progressed in rural regions due to the establishment of micro and small enterprises by individuals endowed with greater financial resources and investment ability. Income disparity may be exacerbated by economic activity and wealth concentration. In rural areas, the non-agricultural sector may experience growth due to increased demand for non-agricultural employment, particularly among low-income households in search of alternative income streams. But access of households to non-agricultural industries may be hindered by challenges related to financing, education, and training. Income disparity may worsen as a result of a concentration of non-agricultural jobs in wealthy households.

### 4.5 Spatial temporal patterns of the gini coefficient

The Global Getis-Ord General Gi* (see Equations (2), (3), (4))) detects income Gini index spatial clusters or patterns. It determines high- and low-value districts by measuring their similarities. The z-score (see Appendix C) reflects the observed *G* value's divergence from the null hypothesis of random spatial distribution. With a *z*-score of −0.235 in 1995, the *G* value is slightly lower than projected. Two positive *z*-scores, 0.093 and 0.546, between 2000 and 2005 indicated a higher *G* value than expected. The *z*-score is −0.256 and −0.013 in 2010 and 2016, respectively, indicating a slightly lower *G* value than expected. All *p*-values are greater than 0.05, and the null hypothesis is accepted. The results suggest that the variable examined over the period has no significant regional clustering or dispersion, and any *G* value variations are likely due to random variation.

**Fig. 5:**
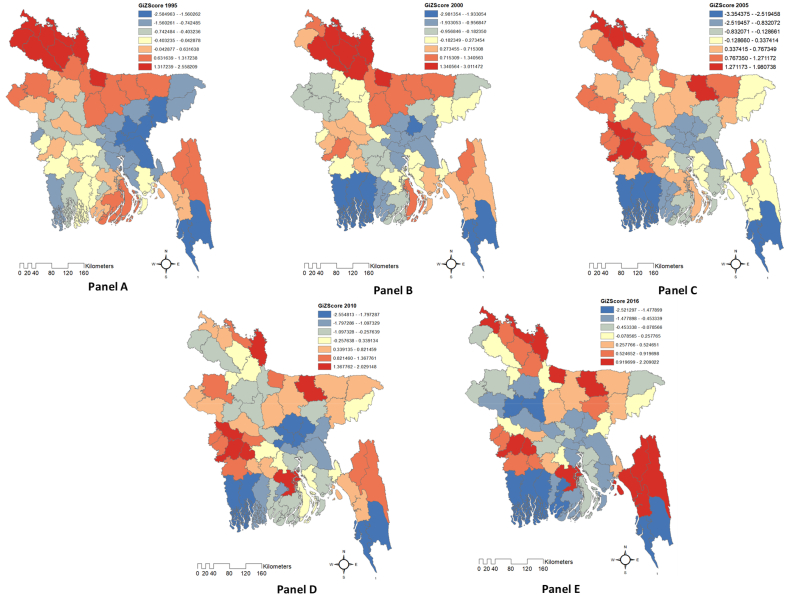
Hot spots of Global Getis-Ord General Gi* of Gini coefficient in Bangladesh.

High- and low-income inequality has been identified using the Getis-Ord Gi* method. The *Z*-score indicates spatial income inequality and positive *Z*-scores imply substantial income inequality in the geographic unit, while negative *Z*-scores indicate low-income inequality. According to Fig. 5, the 1995 (Panel A) and 2000 (Panel B) maps show that high income inequality is centered in the northwestern region particularly in seven districts in the Rangpur division and one district in the Mymensingh division. Greater Rangpur, in northwest Bangladesh, is Monga^2^-affected. Seasonal unemployment and a lack of funds due to the local single-to-two rice crop economy affected female-headed households, agricultural wage laborers, and marginal and small-scale deficit farmers the most during the Monga period [80,81]. This may be one of the reasons for the high-income inequality observed in these districts. Rural households in the northwestern region shifted to a new agricultural pattern due to Monga, focusing on high-value agriculture like fisheries, livestock, poultry, and vegetables. Some households also began engaging in non-farm activities, such as small enterprises and employment in the garment industry. This shift led to a significant increase in RT1 and RT2 after 2000 (Appendix A), causing these districts to transition from a high clustered Gi* score to a low clustered Gi* score. A similar result has been found in northeastern China, where farmers' agricultural income increased due to cash crop cultivation and animal husbandry, and farmers in eastern China had strong entrepreneurship and market knowledge, facilitating income growth [82]. Three districts of the western region exhibited high income inequality in 2005 (Panel C), indicating a hot spot phenomenon. In 2010 (Panel D) and 2016 (Panel E), income inequality spread to the coastal region and the hilly area of the east-southern region. The spatial and temporal distribution of income inequality in Bangladesh has changed. Climate change risks like sea-level rise and cyclones can devastate coastal Bangladeshi economies and worsen income inequality by disproportionately affecting low-income people. In recent years, coastal shrimp aquaculture has grown, which indicates the expansion of RT1. This growth has increased the income of those who are only engaged in shrimp aquaculture, which has led to more income inequality. Hilly areas of Bangladesh are inhabited by several ethnic and linguistic minority groups, where poor transportation, communication, and energy resources limit economic prospects and raise socioeconomic inequality. Tourism in Chattogram's hilly regions may also have triggered income inequality. The growth of the tourism industry in hilly areas has generated new non-agricultural job prospects and boosted the earnings of individuals in this business. This outcome increased RT2 and enhanced the earnings of individuals in the tourism sector, resulting in a rise in income inequality in the region, very similar to northeastern China, where tourism and oil and gas resources led to higher non-agricultural income [82]. In Bangladesh, inequality has re-clustered due to the varied growth of RT1 and RT2 in different districts. This finding is consistent with the research conducted in China, which demonstrated that the spatial and temporal inequalities in rural income inequality are intricately linked to the regional diversification of transition processes involving marketization, globalization, decentralization, and urbanization [83].

### 4.6 Relationship between rural transformation and inequality

To examine the relationship between rural transformation and inequality, we conducted three econometric analyses (see Equations 5-7). Table 3 presents the results of six regression analyses with the Gini index as the dependent variable. Models 1, 3, and 5 illustrate pooled, fixed effect, and non-linear fixed effect results excluding the control variables. Models 2, 4, and 6 illustrate pooled, fixed-effect, and non-linear fixed-effect results, including the control variables. The likelihood ratio tests (p < 0.01) suggest that fixed-effect models are preferred over pooled estimation.

**Table 3:** Pooled and Fixed effects regression results of the Gini index.

	(1)	(2)	(3)	(4)	(5)	(6)
Variables	Pooled	Pooled with controls	Fixed Effect	Fixed Effect with controls	Non-linear Fixed Effect	Non-linear Fixed Effect with controls
RT1	0.317***	0.007	0.222***	0.223***	0.723***	0.772***
	(0.000)	(0.888)	(0.001)	(0.002)	(0.000)	(0.000)
RT1^2^					−1.514***	−1.885***
					(0.002)	(0.000)
RT2	0.215***	0.076***	0.308***	0.245***	0.502***	0.385***
	(0.000)	(0.001)	(0.000)	(0.000)	(0.000)	(0.000)
RT2^2^					−0.277***	−0.202**
					(0.000)	(0.012)
SEE		0.296***		0.146**		0.152**
		(0.000)		(0.043)		(0.022)
SHE		−0.008		0.168		0.247*
		(0.955)		(0.237)		(0.071)
RENT		−0.236***		0.168**		0.026
		(0.000)		(0.013)		(0.686)
SR		0.101***		0.040*		0.010
		(0.000)		(0.069)		(0.635)
GSP		0.757***		−0.189**		−0.056
		(0.000)		(0.022)		(0.471)
_cons	0.280***	0.328***	0.251***	0.254***	0.186***	0.202***
	(0.000)	(0.000)	(0.000)	(0.000)	(0.000)	(0.000)
Observations	320	320	320	320	320	320
R-squared	0.697	0.827	0.927	0.932	0.941	0.943

*Note: p*-values in parentheses *p < 0.1, **p < 0.05, ***p < 0.01.

Table 3 shows that the values of R-squared are high for all regression results (Models 1–6), indicating that a significant proportion of the variability in income inequality is explained by the independent and control variables included in the models. By excluding the control variables (Models 1, 3 and 5), the R-squared values are also high, which implies that RT1 and RT2 provide a strong explanatory power, making the model a better fit to describe the relationship between income inequality and RT. As likelihood ratio tests indicate that fixed-effect regressions are better, we are only interpreting the results of the fixed effects regressions with control variables. Model 4 controls for unobserved district heterogeneity that may affect income inequality with fixed effects. Increased high-value agricultural and rural non-farm employment leads to larger income inequality in districts, as shown by the positive and statistically significant coefficients of RT1 (0.223, p-value <0.01) and RT2 (0.245, p-value <0.01). Model 6 estimates a non-linear relationship between RT and the Gini index by adding a square term (${\text{RT}1}^{2}$ and ${\text{RT}2}^{2}$). The coefficients of RT1 and RT2 in this model are positive and statistically significant, indicating that an increase in RT1 and RT2 leads to higher income inequality in a district. High-value agriculture has a larger market value than cereals, yet cereal production has lower price volatility than high-value agricultural commodities. High-value agricultural producers earn more by minimizing price volatility, increasing the income difference between cereal and high-value agriculture producers [69]. Involvement in high-value agriculture might increase revenues, but it also involves additional financial, economic, and environmental hazards. If not appropriately controlled, these hazards might exacerbate income inequality within communities [84,85]. Another reason for increasing income inequality due to RT1 is that market-oriented expansion of production in rural areas can limit female ownership over income earned from high-value agriculture by imposing constraints on their ability to access markets, particularly impacting their income from yard gardening. This could lead to a rise in income inequality [86]. The rise in non-farm income due to RT2 is driven by wealthy households earning wages outside of agriculture, which has negative distributional impacts. A trend toward non-farm specialization has reduced income from mixed sources (farm and nonfarm) and increased income from non-farm activities, increasing the income gap between these two groups [87]. The increase in non-farm income has exacerbated income inequality in rural households in Bangladesh because adults in rural regions have inadequate skills, which restricts their ability to access non-farm employment and increases the income disparity between individuals engaged in farming and non-farming activities [69,88]. Another reason is that urban proximity increases the likelihood of high-paying non-agricultural jobs. In contrast, people living in remote areas, especially in agricultural areas, are less likely to find well-paying jobs outside of farming, creating an income gap between the two sectors [89]. The coefficients of ${\text{RT}1}^{2}$ (−1.885, p-value <0.01) and ${\text{RT}2}^{2}$ (−0.202, p-value <0.05) are negative and statistically significant, suggesting that the relationship between high-value agriculture and the share of rural non-farm employment and the Gini index is nonlinear, with a positive effect at lower levels and a negative effect at higher levels of RT1 and RT2. The relationship between rural transformation and income inequality is an inverted U shape. According to Kuznets’ hypothesis [90], it suggests that during the development of a nation, inequality increases at first and then decreases in tandem with the growth of rural transformation. A positive and significant coefficient exists for household education expenditure (SEE) in Models 4 (0.146, p-value <0.05) and 6 (0.152, p-value <0.05), demonstrating that increased education investment by households increases income inequality. In both high- and low-income countries, empirical evidence suggests that education is positively correlated with income inequality, whereas in middle-income nations, education is negatively correlated with income inequality [46,48]. The effect of the share of household healthcare expenditure (SHE) is not significant in any model except for Model 6, where it has a positive coefficient of 0.247 (p-value <0.1). The distribution of disposable income in Bangladesh tends to favor the rich due to healthcare costs. The post-payment disposable income of the poor falls as the income of the rich rises. This phenomenon leads to an increase in socio-economic disparity [91].

Farmland rental activity (RENT) has a positive effect on income inequality in Model 4, with a coefficient of −0.168 (p-value <0.05). This result is consistent with another study, where Geng et al. [92] found that the involvement of households in the agricultural rental market in China resulted in a significant increase in rent income for those who rent in and a decrease in rent income for those who rent out, ultimately leading to a widening of the income gap between the two groups. The share of remittance (SR) has a positive effect on the Gini index in Model 4 with a coefficient of 0.040 (p-values <0.01). Remittances initially exacerbate income inequality in Bangladesh because migrant households earn more. A substantial portion of remittances is utilized for consumption, thereby exacerbating the income inequality between recipients and non-recipients. Over time, however, remittances can reduce income inequality by supporting capital accumulation, productive investments, and human capital development [93]. In Model 4, the government's social safety net program (GSP) has a negative coefficient of −0.189 (p-values <0.05), which indicates that an increase in GSP is associated with a decrease in the Gini index. Government social investment has a favorable impact on income inequality, according to the results. When the government spends more on social programs, income disparity declines. Government social spending has been demonstrated to be more effective than education investment at reducing income inequality [94].

In addition to the usual fixed effect model, MM-QR with fixed effects (see Equation (8)) handles distributional and unobserved heterogeneity in panel data at different quantiles [68,95,96]. This robust method controls outliers and does not make distribution assumptions. We analyzed MM-QR regression results at the 10th, 25th, 50th, 75th, and 90th quantiles.

The MM-QR results (Table 4) indicate that both RT1 and RT2 consistently exert a substantial positive effect on rural income inequality across all quantiles. At higher quantiles, the effect of RT2 diminishes, whereas the effect of RT1 remains constant across all quantiles. This finding indicates that RT1 consistently has significant positive effects on income inequality, irrespective of the quantile. Additionally, SR, SEE, and RENT all have a positive effect on income inequality, but the level of significance varies across quantiles. SHE increases income inequality at all quantiles, but the effect is not statistically significant. Only GSP exhibits a negative effect on income inequality, which is statistically significant at the 10th, 25th, and 50th percentiles. Therefore, the effect of rural transformation on rural income inequality is more pronounced than that of other control variables, and it is evident from the results that rural transformation in Bangladesh requires greater attention to reduce rural income inequality over the long term.

**Table 4:** MM-QR with fixed effects as a result of the Gini index.

	FE	FE	FE	FE	FE
Variables	(τ = 0.1)	(τ = 0.25)	(τ = 0.5)	(τ = 0.75)	(τ = 0.9)
RT1	0.223*	0.223**	0.223***	0.223**	0.223*
	(0.088)	(0.021)	(0.001)	(0.019)	(0.089)
RT2	0.260***	0.254***	0.245***	0.236***	0.230***
	(0.000)	(0.000)	(0.000)	(0.000)	(0.000)
SEE	0.291	0.234*	0.143	0.059	−0.003
	(0.106)	(0.081)	(0.142)	(0.656)	(0.988)
SHE	0.085	0.117	0.169	0.217	0.252
	(0.722)	(0.508)	(0.186)	(0.214)	(0.295)
RENT	0.172	0.170**	0.168***	0.166**	0.164
	(0.136)	(0.046)	(0.006)	(0.049)	(0.157)
SR	0.023	0.029	0.040*	0.050*	0.057
	(0.565)	(0.317)	(0.059)	(0.086)	(0.154)
GSP	−0.263*	−0.234**	−0.188**	−0.146	−0.115
	(0.073)	(0.032)	(0.017)	(0.174)	(0.438)
*N*	320	320	320	320	320

*Note: p*-values in parentheses * *p* < 0.1, **p < 0.05, ***p < 0.01.

## 5 Conclusion

Regional income inequality in rural Bangladesh has been rising since the 1990s. This is a cause for concern, particularly in the context of social stability and unity with minority groups who are primarily residing in less developed regions. This study demonstrates that income inequality is most obvious between the 10 %–70 % segment of the population. In rural areas, higher income percentiles are expanding as salaries and remittances from abroad become the primary sources of non-farm income. This indicates the need for policies to reduce the income gap between 10 % and 70 % segment of the population. For example, a safety net for poor and ultra poor people might be provided through strengthening and expanding social safety net programs such as welfare, food assistance, and unemployment benefits.

In this study, RT1 and RT2 initially raised income disparity, but the non-linear relationship shows that RT will diminish it over time. Equal access to resources in rural areas is essential for rural transformation to boost rural income in Bangladesh. The government might encourage high-value agriculture by facilitating small-scale farmers’ access to land, water, and fertilizer. Implementing credit programs for small-scale producers and regulating resource concentration in a few large proprietors or businesses can achieve the goal of equitable natural resource allocation in rural areas. In particular, the government should establish price adjustment policies to reduce the price volatility of high-value agricultural products, provide storage facilities for perishable high-value agricultural products, and consider the needs of specific rural areas in order to reduce income inequality caused by RT1. Assistance could be provided through the establishment of special loan programs for individuals who are unable to participate in high-value agriculture due to financial constraints, the provision of subsidized inputs, including high-value agricultural seeds, fertilizers, and other materials, and the execution of special arrangements with suppliers of agricultural machinery. Youth engagement in high-value agriculture could be encouraged through the implementation of training programs at the village level. To mitigate income inequality due to RT2, the government could consider establishing specialized programs to support financially disadvantaged rural residents for alternative income sources. Priority should be given to small-scale industrialization in rural areas utilizing locally produced goods, and opportunities for these goods to be sold on a national and international level should be created. In order to restructure the wage system, the government could reduce the wage gap by providing investments in education to improve the education and skills of every individual.

In addition to RT1 and RT2, this study demonstrates that agricultural rental systems, remittances, and household educational expenditures contribute to income inequality in rural Bangladesh. The government may regulate and standardize agricultural rental agreements to ensure fair terms for landowners and tenants, establish government-backed platforms or local committees to regulate rental arrangements, setting clear lease durations, rent levels, and dispute settlement. Policymakers may collaborate with financial institutions to encourage remittance receivers to invest in productive sectors like small-scale industries in economically vulnerable rural areas rather than immediate consumption. For example, providing tax advantages will encourage remittance-funded investments in certain sectors. Policies to promote financial literacy through small business, agricultural, and education investments would also be beneficial. The government should also provide targeted, conditional cash transfers to poor rural households suffering from education costs. This support must have clear eligibility criteria and a transparent application process to help families with financial restrictions to send their children to school.

Finally, this study also uncovered the positive effects of government social safety net programs on income inequality among rural households. The government of Bangladesh provides four different categories of social safety net programs: monetary transfers, food security and employment generation programs, stipend programs, and cash or material transfers. These initiatives have the potential to reduce rural income inequality, but there needs to be greater transparency in how they are implemented. The government may implement spatial mapping for economically vulnerable areas and establish an online database for people experiencing financial difficulties so that people registered to receive social security assistance can receive social safety support directly by the most direct means, e.g., through a mobile banking system in order to ensure efficiency and transparency. The funding for this program should be based on the particular rural area's economic situation, for example, by allocating a larger budget to residents of places that have experienced more natural disasters. These initiatives have the potential to enhance economic sustainability and equity in Bangladesh, thereby mitigating rural income inequality. Finally, our research results provide valuable insights for policymakers and academics in agrarian economies where RT is still in the early stages and will provide valuable information on inclusive strategies to address income inequality in rural settings globally.

## Data availability

https://dataverse.harvard.edu/dataset.xhtml?persistentId=doi:10.7910/DVN/OZQGNV.

## CRediT authorship contribution statement

**Al Amin Al Abbasi:** Writing – original draft, Methodology, Formal analysis, Data curation, Conceptualization. **Subrata Saha:** Writing – original draft, Formal analysis, Data curation, Conceptualization. **Ismat Ara Begum:** Writing – review & editing, Validation, Supervision, Methodology. **Maria Fay Rola-Rubzen:** Writing – review & editing, Validation, Supervision, Conceptualization. **Andrew M. McKenzie:** Writing – review & editing, Visualization, Validation, Methodology. **Mohammad Jahangir Alam:** Writing – review & editing, Validation, Supervision, Project administration, Methodology, Funding acquisition, Data curation, Conceptualization.

## Declaration of competing interest

The authors declare that they have no known competing financial interests or personal relationships that could have appeared to influence the work reported in this paper.

## Footnotes

1 The eastern districts of the country are surrounded by water, known as the Haor area [97].

2 The local name "Monga" is most frequently used to refer to a famine-like scenario that occurs twice a year: first during the Bengali months of Ashwin-Kartik (middle of September to middle of November), and again during Chaittra-Baishak (middle of March to middle of May) [80].
