# Supplementary material for: Does rural transformation affect rural income inequality? Insights from cross-district panel data analysis in Bangladesh
Source: Heliyon. 2024 Apr 30;10(9):e30562. doi: 10.1016/j.heliyon.2024.e30562 (PMC11079257; doi:10.1016/j.heliyon.2024.e30562)
Supplement: Multimedia component 1 [file mmc1.docx]

1. Trends of RT1, RT2, and Gini index


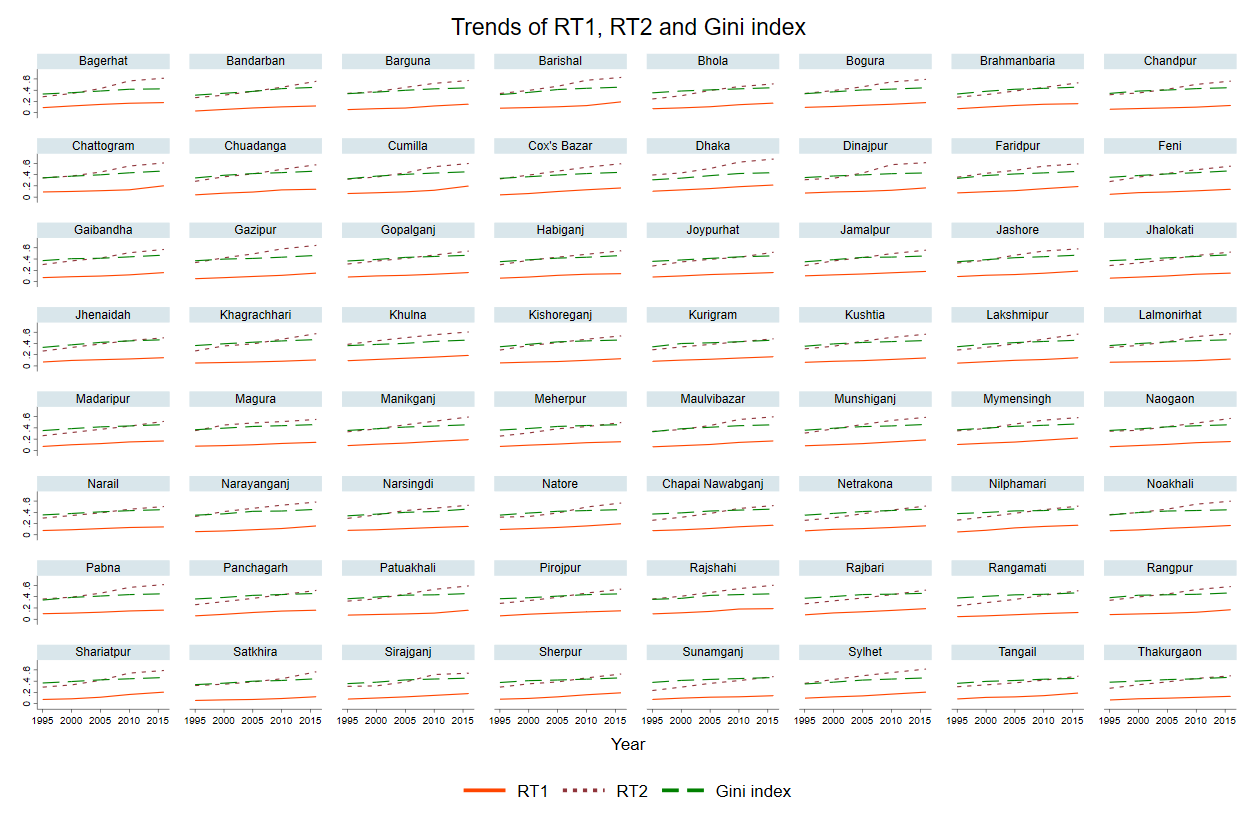


1. Correlation between Gini index and rural transformation


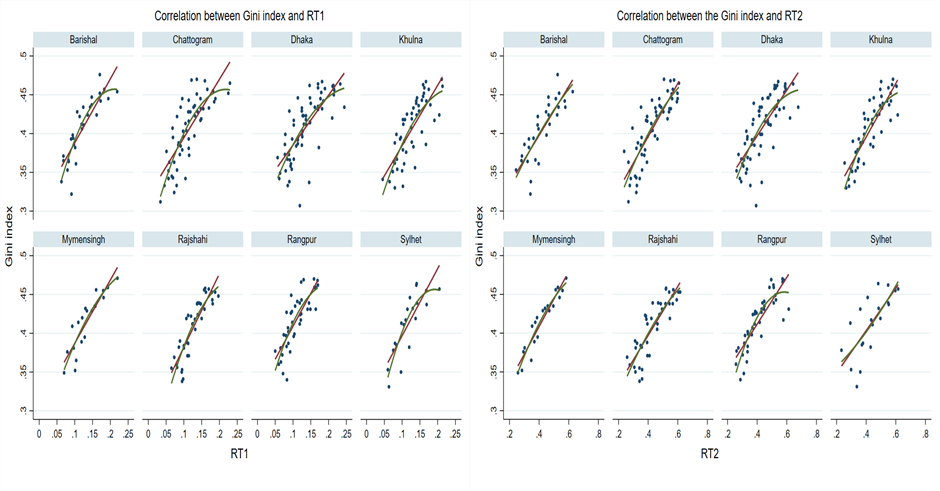


Appendix C Global Getis-Ord General Gi* of Gini coefficient

| Year | 1995 | 2000 | 2005 | 2010 | 2016 |
| --- | --- | --- | --- | --- | --- |
| Observed G | 0.067 | 0.067 | 0.067 | 0.067 | 0.067 |
| z-score | -0.235 | 0.093 | 0.546 | -0.256 | -0.013 |
| p-value | 0.814 | 0.926 | 0.585 | 0.798 | 0.990 |
